# Correction to “The challenges of growing orchids from seeds for conservation: An assessment of asymbiotic techniques”

**DOI:** 10.1002/aps3.70079

**Published:** 2026-08-25

Jolman, D., M. I. Batalla, A. Hungerford, P. Norwood, N. Tait, and L. E. Wallace. 2022. The challenges of growing orchids from seeds for conservation: An assessment of asymbiotic techniques. *Applications in Plant Sciences* 10(5): e11496. https://doi.org/10.1002/aps3.11496

In the original publication of this article, an error occurred in the formatting of the name of the author Dr. Jaime A. Teixeira da Silva in a cited reference.

The corrected reference should read:

Fu, Y. Y., N. Jiang, K. L. Wu, J. X. Zhang, J. A. Teixeira da Silva, J. Duan, H. T. Liu, and S. J. Zeng. 2016. Stimulatory effects of sodium hypochlorite and ultrasonic treatments on tetrazolium staining and seed germination in vitro of *Paphiopedilum* SCBG Red Jewel. *Seed Science and Technology* 44: 77–90. https://doi.org/10.15258/sst.2016.44.1.13

We apologize for this error.

